# Comparative transcriptome analysis reveals the biocontrol mechanism of *Bacillus velezensis* E68 against *Fusarium graminearum* DAOMC 180378, the causal agent of Fusarium head blight

**DOI:** 10.1371/journal.pone.0277983

**Published:** 2023-01-26

**Authors:** Nathan Liang, Jean-Benoît Charron, Suha Jabaji

**Affiliations:** Department of Plant Science, Faculty of Agricultural and Environmental Sciences, McGill University, Montreal, Quebec, Canada; Newcastle University, UNITED KINGDOM

## Abstract

*Fusarium graminearum* is the causal agent of Fusarium Head Blight, a serious disease affecting grain crops worldwide. Biological control involves the use of microorganisms to combat plant pathogens such as *F*. *graminearum*. Strains of *Bacillus velezensis* are common biological control candidates for use against *F*. *graminearum* and other plant pathogens, as they can secrete antifungal secondary metabolites. Here we study the interaction between *B*. *velezensis* E68 and *F*. *graminearum* DAOMC 180378 by employing a dual RNA-seq approach to assess the transcriptional changes in both organisms. In dual culture, *B*. *velezensis* up-regulated genes related to sporulation and phosphate stress and down-regulated genes related to secondary metabolism, biofilm formation and the tricarboxylic acid cycle. *F*. *graminearum* up-regulated genes encoding for killer protein 4-like proteins and genes relating to heavy metal tolerance, and down-regulated genes relating to trichothecene biosynthesis and phenol metabolism. This study provides insight into the molecular mechanisms involved in the interaction between a biocontrol bacterium and a phytopathogenic fungus.

## Introduction

*Fusarium graminearum* is the causal agent of Fusarium Head Blight (FHB) in wheat and barley [1]. It is considered a major global threat that impacted great economic losses on the cereal industry because of reduced grain yield and grain quality. FHB can also cause contamination of crops and grains with diverse mycotoxins, including deoxynivalenol (DON) and zearalenone, which are harmful for humans and animals [2, 3]. FHB has severe impacts on grain yield, with reported losses of up to 80% [4]. It has been estimated that in 2015, FHB costed the US wheat and barley industries $1.469 billion in value of yield forgone [5]. Recent data for Canada is not available, though it has been estimated that $520 million were lost by wheat producers in Ontario, Quebec and Manitoba during the 1990s [6]. Grain quality is affected as well, as kernels become discoloured, damaged and light in weight. Additionally, *Fusarium* may secrete mycotoxins into the crops. Contaminated grain can have adverse health effects when consumed, whether by humans or livestock. Contaminated grain can also lead to reduced seed germination and vigour [7]. Due to these factors, market price for affected grain becomes severely discounted.

Control of FHB requires a combination of multiple management strategies, including a well-thought out crop rotation strategy and the use of resistant crop cultivars. Another management strategy for FHB is the use of chemical fungicides. Triazole fungicides inhibit the cytochrome P450 sterol 14α-demethylase, and consequently, ergosterol biosynthesis. Triazoles could reduce disease index (diseased spikelets per spike) by 52%, and reduce DON concentration by 45% [8]. However, it has been shown that fungicides become much more effective when used on cultivars with at least moderate resistance to FHB [9]. Triazole residues have been detected in many locations, from wastewater, to soil, to human hair and urine samples. Evidence shows that triazoles can lead to endocrine disruption, hepatotoxicity, cytotoxicity and liver carcinogenesis [10]. In addition, chemical fungicides are quite expensive and may lead to farmers choosing not to produce FHB-susceptible crops [5]. Concerns have emerged as numerous strains of *F*. *graminearum* have been identified with resistance to triazoles [11–13]. As a result, there is a need for an alternative to these fungicides.

A common tactic for phytopathogen management is the use of biological control methods. This implies the use of living organisms (viruses, bacteria, fungi, insects, etc.) to suppress or eliminate the growth of plant pests. The use of microorganisms is most common, whether employing them live, or extracting their antimicrobial metabolites. Microorganisms offer a distinct advantage in that their mass production and application is very convenient. Many different mechanisms of action are possible. The microorganisms may induce systemic resistance in the plant without direct interaction with the pathogen, compete for nutrients and space, parasitize or antagonize the pathogen or secrete antimicrobial compounds which inhibit the pathogen (antibiosis). Alternatively, the microorganisms may deal specifically with mycotoxins, and sequester or degrade them in some fashion [14].

Biological control methods tend to offer less disadvantages than chemical control, as there are many less health risks to the consumer, and no risks of environmental contamination. Additionally, biological control is usually narrower in scope, avoiding non-specific effects to other non-target organisms. Isolates of *Trichoderma harzianum* Rifai reduced *F*. *graminearum* perithecial formation on wheat straw residues in the field and might have the potential to reduce inoculum development [15]. Compared to the registered fungicide Folicur, a strain of the fungus *Clonostachys rosea* reduced infected spikelets by 58%, and reduced *Fusarium* damaged kernels by 49% and DON by 21%. These effects were significant but of lesser magnitude than those achieved by the tebuconazole fungicide [16]. Other effective microorganisms include species of *Pseudomonas*, *B acillus*, *Streptomyces* and yeasts [17, 18]. These originated from various sources such as sourdough bread, peanut shells, soil and wheat itself. These biocontrol agents have been tested both *in vitro* and in greenhouse/field experiments. Biocontrol in FHB specifically has been mediated mostly through 2 mechanisms: either secretion of antifungal compounds or competition for nutrients and space, particularly iron [17].

*Bacillus velezensis* is a bacterial species, member of the *Bacillus subtilis* species complex. Bacteria from this group can be found in several different environments, with some living as plant endophytes [19, 20]. *B*. *velezensis* and its related species can produce and secrete secondary metabolites known as cyclic lipopeptides. These molecules are biosurfactants which have demonstrated antimicrobial ability against fungal phytopathogens in both field studies and *in vitro* [21]. *B*. *velezensis* and molecules derived from it have shown to inhibit growth of *F*. *graminearum in vitro* and limit FHB disease progression *in planta* when applied as biocontrol agents [22–24]. The bacterial strains from these studies and similar ones are typically endophytes isolated directly from plants, ensuring their ability to colonize the hosts. However, it is important to assess the abilities of bacterial strains from other environments as they may have differing effects as biocontrol candidates as compared to endophytes.

*Bacillus velezensis* strain E68 was previously isolated from crude oil samples from the Albertan oil sands [25, 26]. Oil-dwelling bacteria must be adapted to live in the harsh conditions: high temperature, high pressure and low carbon [27, 28]. These bacteria have adapted to the particular organic carbon sources present in these environments, including hydrocarbons. Biosurfactants secreted by these bacteria are used to degrade hydrocarbons for use as a carbon source [29]. Much research has gone into studying the potential of these biosurfactants for bioremediation of hydrocarbon pollutants [30]. However, oil-dwelling bacteria are unexplored when it comes to biocontrol. In preliminary experiments, *B*. *velezensis* E68 was found to inhibit the growth of various fungal phytopathogens such as *Rhizoctonia solani* and *Fusarium graminearum* (S1 Fig). Also, the cell-free supernatant of strain E68 exhibited biosurfactant activity using drop collapse and agar spreading assays [26]. These results suggest strain E68 has a wide inhibition spectrum against plant fungal pathogens.

The main task of the present research is to dissect the undergoing changes in the transcriptome profiles of both partners following dual culture of *B*. *velezensis* E68 and *F*. *graminearum* DAOMC 180378 using RNA-seq. This could provide valuable insights into the main factors that determine its outcome. Using the RNA-seq method, we observed the transcriptional activity of both organisms in response to the presence of one another.

## Methods

### Biological material and culture conditions

Cultures of *Fusarium graminearum* strain DAOMC 180378 were provided by the Canadian Collection of Fungal Cultures (DAOMC; Agriculture and Agri-Food Canada). *Bacillus velezensis* strain E68 was previously isolated from an oil well in Alberta [25, 26]. Microbial strains were retrieved from 20% glycerol stock stored at -80°C. *F*. *graminearum* DAOMC 180378 was grown on potato dextrose agar (PDA; Difco Laboratories) at 25°C in the dark. *B*. *velezensis* E68 was grown on Luria-Bertani agar (LBA) at 37°C.

### Inhibitory effects of *Bacillus velezensis* E68 on *Fusarium graminearum* DAOMC 180378 mycelial growth

**Fig 1:**
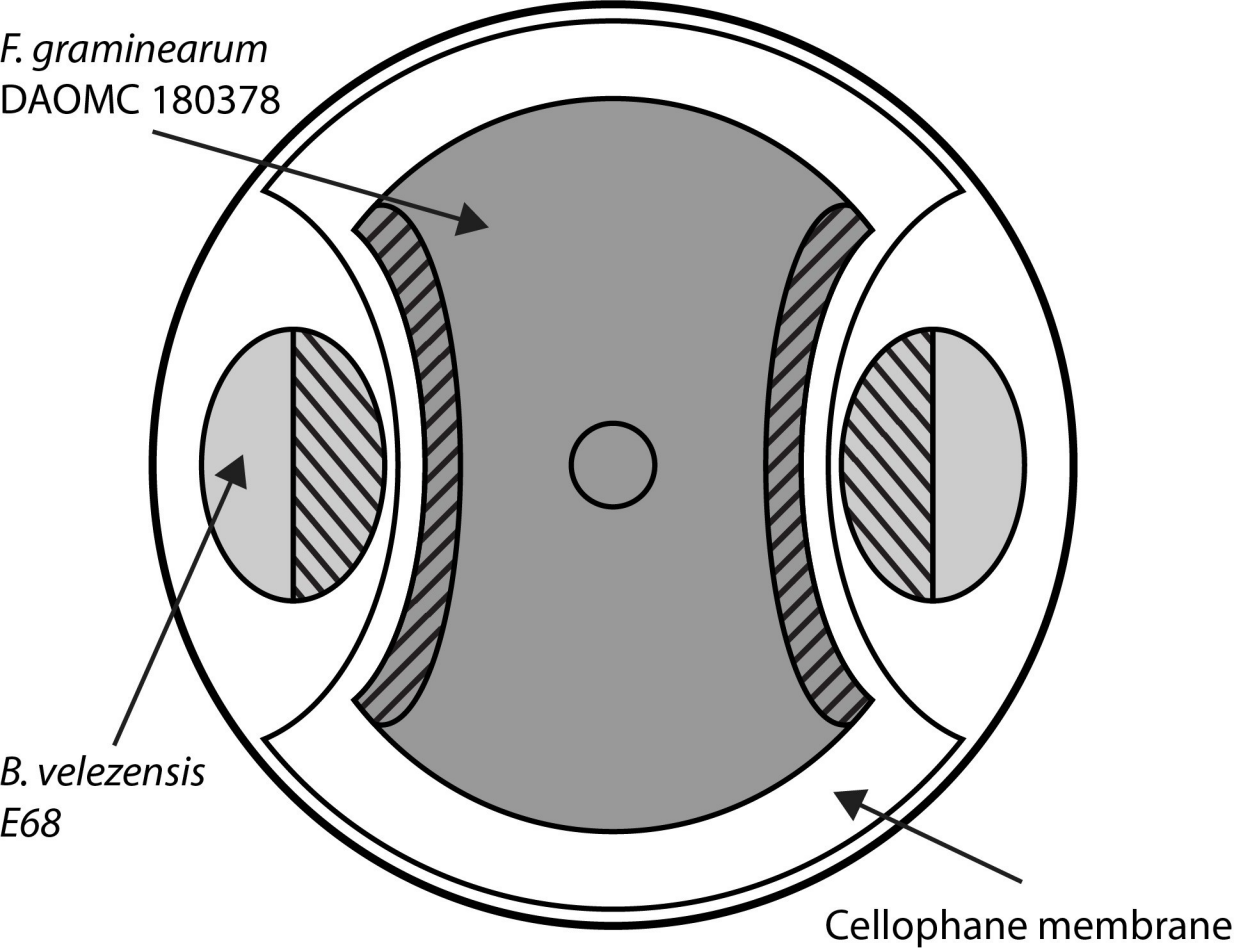
Dual-culture interaction plate setup. *Fusarium graminearum* DAOMC 180378 hyphae (dark grey) was grown from an agar plug placed at the centre of the plate on a cellulose membrane. Colonies of *Bacillus velezensis* E68 (light grey) were grown from aliquots of cell suspension spotted 2.5 cm from the centre of the plate. Striped areas were collected with a sterile loop or spatula for RNA extraction. Diagram is not to scale.

In order to test the inhibitory effect of *Bacillus velezensis* E68 on *Fusarium graminearum* DAOMC 180378 mycelial growth, the two organisms were grown in dual culture. Sterilized circular cellophane membranes (UCB, North Augusta, USA) were overlaid on 9 cm diameter petri plates containing 20 mL of PDA. Two half-moon sections were cut out from opposite sides of the membrane up to 2 cm from its center in order for *B*. *velezensis* to grow directly on the culture media (Fig 1). An agar plug (5 mm diameter) was taken from 7-day old cultures of actively growing *F*. *graminearum*, and were placed at the center of each plate overlaid with cellophane. A 10 μL aliquot of *Bacillus velezensis* (OD_600_ of 1.0) from an overnight LB broth was spotted on 2 opposite edges of the plate where the cellophane membrane was cut out, 2.5 cm from the center of the plate. A 0.5 cm agar plug of *F*. *graminearum* placed at the center of a PDA plate overlaid with a cellophane membrane served as control (Fig 1). Three 10 μL aliquots of *Bacillus velezensis* (OD_600_ of 1.0) spotted apart on a PDA plate served as control (Fig 1). Dual culture and control plates were incubated at 24°C for 3 days before RNA extraction.

Dual culture and control plates were set up for microscopy as described above. A strip of cellophane membrane (0.5 cm x 2 cm) containing edges of the fungal hyphae was carefully excised from both a dual culture and a control treatment plate. The strips were placed on a microscopy slide, stained with bromophenol blue and mounted for microscopy on an EVOS XL Core Imaging System (Invitrogen).

### Sample preparation and RNA extraction

Bacterial colonies were collected with a sterile loop by removing the half of each colony that was facing the center of the plate (Fig 1). In total, 6 halves of bacterial colonies were pooled into one RNA sample (i.e. 3 dual-culture plates pooled into 1 sample and 1 control plate pooled into 1 sample). The bacterial cells was placed into 500 μL of sterile water per sample for immediate extraction. Strips of fungal hyphae (5 x 50 mm) closest to the bacterial spots were collected with a sterile spatula (Fig 1). Six hyphal strips were pooled into one RNA sample. For the control treatment, an equivalent amount of hyphae was taken from the edges of a fungal colony and used as one sample. The fungal material was immediately ground with a mortar and pestle in liquid nitrogen and stored at -80°C before RNA extraction.

High quality total RNA from *B*. *velezensis* and *F*. *graminearum* was extracted using the Nucleospin RNA kit (Takara). RNA extraction from *B*. *velezensis* followed manufacturer’s instructions for Gram-positive bacteria, with modifications: the cell pellet was resuspended in 100 μL TE buffer (10 mM Tris, 1 mM EDTA, pH 8.0) with 3 mg/mL lysozyme, then incubated for 15 minutes at 37°C. RNA extraction from *F*. *graminearum* followed the instructions for cultured cells and tissues. For both organisms, 600 μL of buffer RA1, 6 μL of β-mercaptoethanol and 600 μL of 70% ethanol were used instead of 350 μL, 3.5 μL and 350 μL respectively. In all cases, elution was done by eluting twice in 60 μL of RNase-free water. RNA samples integrity and concentration were assessed by 1% bleach agarose gel and Nanodrop ND-1000. Samples were stored at -80°C for downstream applications.

### RNA sequencing

Five RNA biological replicates for each treatment (*B*. *velezensis* control and dual-culture, *F*. *graminearum* control and dual-culture) were sent for library preparation and sequencing at IDSeq Inc, (Sacramento, California). The *B*. *velezensis* libraries were prepared with a TruSeq RNA library prep kit, with a Ribo-Zero Plus rRNA depletion kit. The *F*. *graminearum* libraries were prepared with the NEBNext Ultra II RNA library prep kit with poly-A selection. Samples were sequenced on an Illumina HiSeq 4000.

### RNA-seq analysis

**Fig 2:**
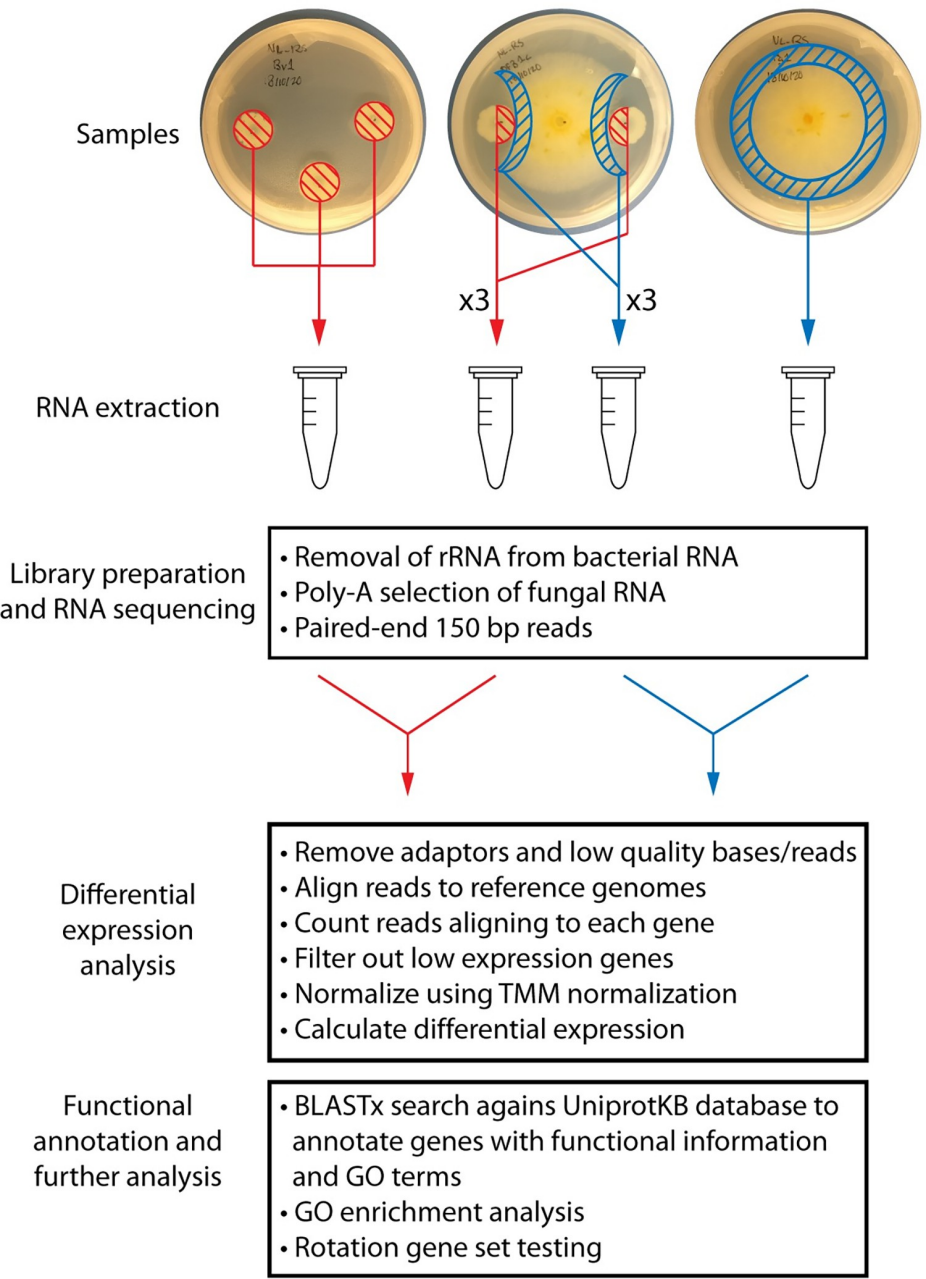
Schematic of experimental workflow of RNA-seq analysis of *Bacillus velezensis* and *Fusarium graminearum*. Cultures of *B*. *velezensis* E68 and *F*. *graminearum* DAOMC 180378 were grown in single and dual culture. The striped areas were collected with a sterile spatula before RNA extraction. Total RNA was sent for library preparation and RNA sequencing. Bacterial RNA was depleted for ribosomal RNA, while fungal RNA was enriched with poly-A selection. Samples were sequenced on an Illumina HiSeq using a paired-end 150 bp protocol. Raw sequencing reads were pre-processed by removing adaptors and poor quality bases and reads. Filtered reads were aligned to their respective genomes and counted for each gene. Gene counts were normalized by the TMM method, low expression genes were removed from analysis and differential expression was calculated. In order to functionally annotate the genes, each gene sequence was used in a BLASTx search to compare to the UniprotKB protein database, annotating each gene with a description and gene ontology terms. The resulting GO terms were used in GO enrichment analysis. Known biosynthetic gene clusters were used in rotation gene set testing to determine their overall regulation pattern.

Fig 2 depicts the pipeline followed from sample preparation to RNA-seq data analysis. Sequencing reads were checked for quality and adaptor contamination before and after trimming with FastQC 0.11.9 [31]. Low quality bases and adaptor sequences were trimmed from the raw sequencing reads with Trim Galore 0.6.5 with stringency set to 3 [32]. The genome assembly for *F*. *graminearum* DAOMC 180378 is available on GenBank, however no reference annotation is available. The genome annotation version 5 [33] from *Fusarium graminearum* PH-1 was transferred to the genome assembly of DAOMC 180378 using Liftoff 1.6.1 [34]. The resulting annotation was converted from GFF3 format to GTF format with gffread 0.12.3 [35]. Trimmed reads were mapped to the reference genomes for *F*. *graminearum* DAOMC180378 and *Bacillus velezensis* E68 using STAR 2.7.5a on default settings [25, 36, 37]. The GeneCounts function from STAR was used to obtain read counts. Differential gene expression was assessed using edgeR 3.32.1 [38]. Genes were filtered by CPM expression using edgeR default settings. The edgeR GLM framework was used. Genes with a log_2_ fold change under 1.5 were deemed non-significant using the glmTreat function. Genes with a p-value under 0.05 after correction for multiple testing were deemed significant and differentially expressed. edgeR was also used to perform rotation gene set testing (ROAST) [39]. Genes were functionally annotated with gene ontology data by using Trinotate 3.2.2 to facilitate BLASTx searches of the gene coding sequences against the UniProtKB protein database [40]. Gene ontology enrichment analysis was carried out with GOSeq 1.44.0 to detect enriched gene ontology terms among up and down-regulated genes in both organisms, using a false discovery rate < 0.05 [41]. Biosynthetic gene clusters in *B*. *velezensis* E68 were identified with antiSMASH in previous work [25].

### Validation of RNA-seq by qRT-PCR

In order to confirm the differential expression results obtained by RNA-seq, 8 genes for each organism were selected for validation by qRT-PCR (Table 1). Three RNA samples from each condition were used to validate the RNA-seq. The RNA samples were transcribed into cDNA using the Agilent AffinityScript qPCR cDNA Synthesis Kit. Specific primers for the selected genes were designed using Geneious 8.1.9 and the OligoAnalyzer tool from IDT. Primers are listed in Table 1. Specificity was assessed with PCR and agarose gel. *recA* (GYA98_RS07520) and *secA* (GYA98_RS15380) were used as housekeeping genes for *B*. *velezensis* [42]. *GzUBH*, ubiquitin thiolesterase (FGRAMPH1_01G03049) and *TUB2*, tubulin beta chain (FGRAMPH1_01G26865) were used as housekeeping genes for *F*. *graminearum* [43, 44]. Dilutions of pooled cDNA were used to create standard curves for each primer to calculate reaction efficiency and determine the optimal cDNA concentration for final usage. The geometric mean of the housekeeping genes was used as a normalization factor. The reaction efficiency and normalization factor were used to adjust the ΔC_q_ values of the genes of interest as described in Taylor et al. [45]. Log transformed normalized expression values were compared between single and dual culture using a t-test. Three technical replicates were run per sample.

**Table 1:** List of primers used in this study.

Target locus ID	Gene product, Gene symbol	Primer ID	Sequence (5’– 3’)	Source
Primers for use with *Bacillus velezensis* E68				
GYA98_RS07520^$^	Multifunctional protein RecA	recA-F	AAAAAACAAAGTCGCTCCTCCG	[42]
	*recA*	recA-R	CGATATCCAGTTCAGTTCCAAG	
GYA98_RS15380^$^	Protein translocase subunit SecA	oNL25_secA_f	CGTTTAAAGTGCAGCTGATGG	This paper
	*secA*	oNL26_secA_r	GGCTCGCCAGATATTCGTTG	
GYA98_RS15140	Heme response regulator HssR	oNL45_hssR_F	TATGAAGGTGACGAGCGTAC	This paper
	*hssR*	oNL46_hssR_R	TGGATGGCAAACGGATAGTC	
GYA98_RS00430	Alkaline phosphatase D	oNL47_phoD_F	GCGTTTGAAGTGAATGCCG	This paper
	*phoD*	oNL48_phoD_R	CAGAGAATGACGCTGTCAGAC	
GYA98_RS07565	Spore coat protein E	oNL49_cotE_F	ACCAACACGATCTCACCTGG	This paper
	*cotE*	oNL50_cotE_R	TCGGCGTAAGAGTACCAGAC	
GYA98_RS00125	ECF RNA polymerase sigma factor SigW	oNL53_sigW_F	GCCTGACAAGTACAGAACGG	This paper
	*sigW*	oNL54_sigW_R	CTGCCTCTGTGTATCCTTGTC	
GYA98_RS03850	ECF RNA polymerase sigma factor SigM	oNL67_sigM_F	GACCACAGCAAAGTAAAGCC	This paper
	*sigM*	oNL68_sigM_R	ATACCCTGTCAGCACTTCC	
GYA98_RS15180	ATP phosphoribosyltransferase	oNL69_hisG_F	AGTTATTTCAGAGAGCAGGGC	This paper
	*hisG*	oNL70_hisG_R	CTACGATTCTGTCAGCAAGCC	
GYA98_RS03165	Catechol-2,3-dioxygenase	oNL71_catE_F	GTCATCTCGCAGACAGATCG	This paper
	*catE*	oNL72_catE_R	GGTCAGGCAGAAGTATCGC	
GYA98_RS12240	Citrate synthase 2	oNL73_citZ_F	TGAGATGTCAATCCGTGTCG	This paper
	*citZ*	oNL74_citZ_R	GATCGGCGTGAACAGATCG	
Primers for use with *Fusarium graminearum* DAOMC 180378				
FGRAMPH1_01G03049^#^	Ubiquitin thiolesterase	oNL29_GzUBH-F	CTCGAGGCCAGCAAAAAGTCA	[43]
	*UBH*	oNL30_GzUBH-R	ATCGCCGTTAGGGGTGTCTG	
FGRAMPH1_01G26865^#^	Tubulin beta chain	oNL43_TUB2-F	GTTGATCTCCAAGATCCGTG	[44]
	*TUB2*	oNL44_TUB2-R	CATGCAAATGTCGTAGAGGG	
FGRAMPH1_01G01687	Pleiotropic ABC efflux transporter of multiple drugs YBT1	oNL55_ybt1_F	GCTACGCCTGCAAATGAACC	This paper
	*YBT1*	oNL56_ybt1_R	CTTAGGTCGAGCTACAATGGC	
FGRAMPH1_01G15627	ZEB2-regulated ABC transporter 1	oNL57_zra1_F	GCTTCCAGGACATGAACGTC	This paper
	*ZRA1*	oNL58_zra1_R	GTCAATTCTGCGCTTGTTGG	
FGRAMPH1_01G00143	Nonribosomal peptide synthetase GRA1	oNL59_gra1_F	ACTTGGAAGCTTTCTACCTGG	This paper
	*GRA1*	oNL60_gra1_R	CGTTGATCTATCACCAGCGAG	
FGRAMPH1_01G05661	Acetylesterase	oNL61_aes1_F	TGGTCAAGTCGATCAACCTC	This paper
	*AES1*	oNL62_aes1_R	CAGTGGGATTCTTGATTGCC	
FGRAMPH1_01G12231	D-malate dehydrogenase	oNL63_dmlA_F	GGTAATGACGAACCTGAGCC	This paper
	*DmlA*	oNL64_dmlA_R	CGATGGTGACTGCATGAAGG	
FGRAMPH1_01G05741	Mitochondrial aldehyde dehydrogenase	oNL65_aldh_F	GGAGGAAATCTTTGGACCGG	This paper
	*ALDH2B4*	oNL66_aldh_R	GAGTTGAGCTTCTGAGTGACC	
FGRAMPH1_01G19889	RuvB-like helicase 2	oNL75_RVB2_F	TTCGCAACTTGTCTCTGCC	This paper
	*RVB2*	oNL76_RVB2_R	GGCTACAAACTTGATGCTGC	
FGRAMPH1_01G06539	DNA repair protein	oNL77_RAD5_F	AGTTCACATCTTTCCTCAGCC	This paper
	*RAD5*	oNL78_RAD5_R	CGTTAAGTACAGCGGCTCG	

^$^Housekeeping genes for *Bacillus velezensis* E68.

^#^Housekeeping genes for *F*. *graminearum* DAOMC 180378

## Results

### Dual RNA-seq analysis of *B*. *velezensis* and *F*. *graminearum*

**Fig 3:**
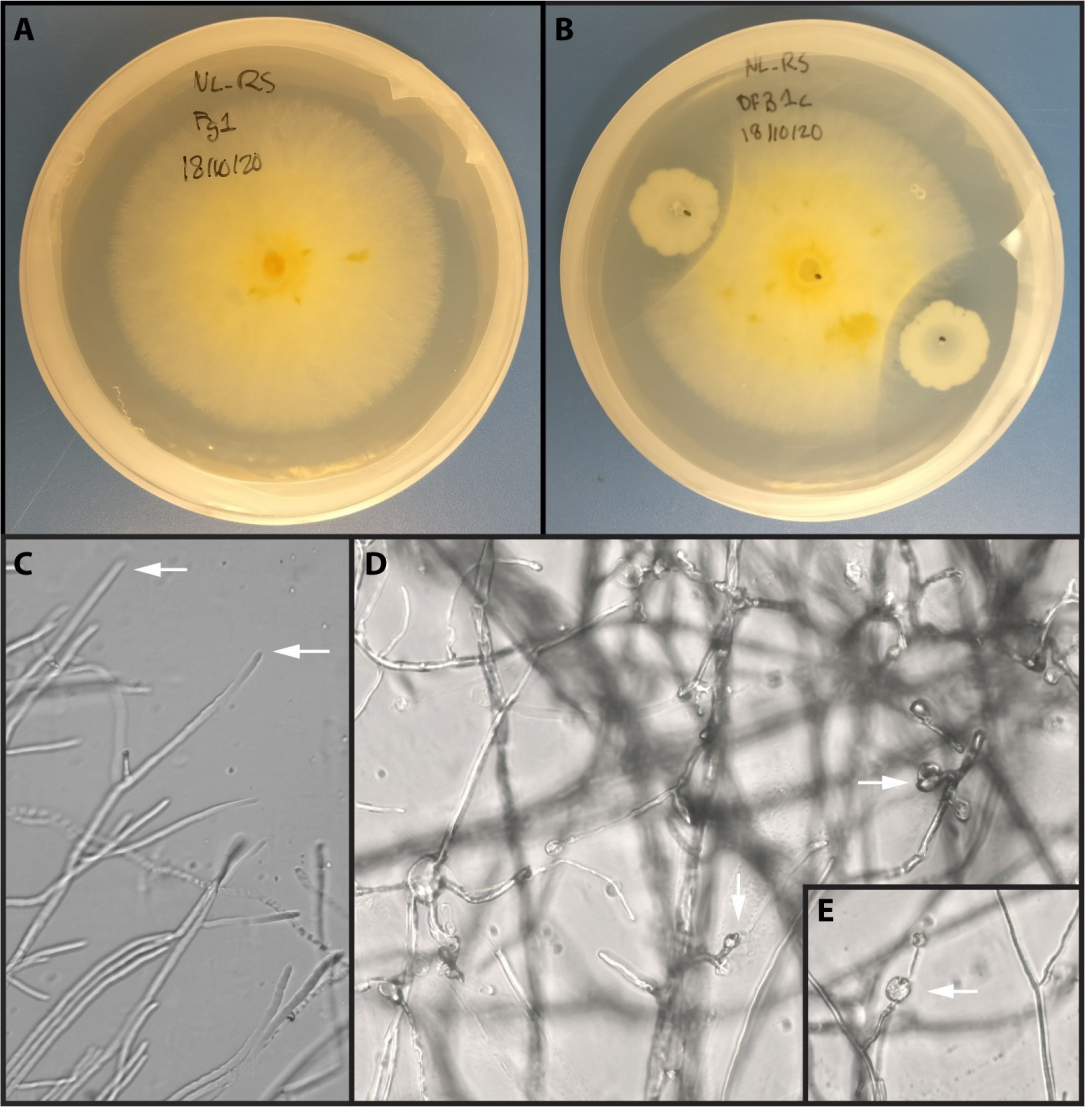
*B*. *velezensis* E68 inhibits the growth of *F*. *graminearum* DAOMC 180378 in dual culture. (A) *F*. *graminearum* DAOMC 180378 grown in single culture on PDA, (B) Dual culture condition for *B*. *velezensis* E68 and *F*. *graminearum* DAOMC 180378 on PDA. (C) Hyphal morphology of *F*. *graminearum* in single culture. (D) and (E) Hyphal morphology of *F*. *graminearum* in dual culture with *B*. *velezensis*. Microscopy performed at 40x magnification.

Cultures of *Bacillus velezensis* E68 and *Fusarium graminearum* DAOMC 180378 were grown together on potato dextrose agar as described in the Methods. Following 3 days of incubation, a clear inhibition of fungal growth was observed (Fig 3B). The morphology of *F*. *graminearum* appeared healthy in monoculture (Fig 3C). When grown in dual culture with *B*. *velezensis*, the morphology of *F*. *graminearum* appeared collapsed, with hyphal tips disrupted and developing circular spheres (Fig 3D and 3E). To evaluate the changes in total gene expression in both organisms, RNA was separately extracted from both organisms growing alone in single and together in dual culture. RNA from both organisms from both conditions was sequenced. After adaptor trimming and filtering the sequencing reads for quality, between 2.9 and 10.4 million sequencing reads were obtained for *B*. *velezensis* samples and between 16.4 and 24.5 million reads were obtained for *F*. *graminearum* samples (Table 2). The remaining reads were aligned to the reference genomes and reads aligning to each gene were quantified. The R package edgeR was used to filter out lowly expressed genes, normalize the libraries and calculate differential expression for each gene between single and dual culture. A total of 168 genes from *B*. *velezensis* and 4207 genes from *F*. *graminearum* were filtered out due to low expression. After calculation of differential expression, genes with a log_2_ fold change under 1.5 between conditions were not considered significant, as well as genes with a false discovery rate above 0.05. With these requirements, 1155 genes (32.4% of all expressed genes) were differentially expressed in *B*. *velezensis* between single and dual culture. In *F*. *graminearum*, 1503 genes (15.3% of all expressed genes) were differentially expressed (Table 3). Of the differentially expressed genes (DEGs), 47.2% were down-regulated in *B*. *velezensis*, whereas 66.1% of DEGs were down-regulated in *F*. *graminearum*. Genes were functionally annotated by BLASTx searches to the UniprotKB database, which also annotated many genes with gene ontology (GO) terms. In *B*. *velezensis*, 2394 genes were functionally annotated with GO terms (67.2% of all expressed genes), while 5464 genes were functionally annotated with GO terms in *F*. *graminearum* (55.6% of all expressed genes).

**Table 2:** Number of sequencing reads from each sample after trimming.

Sample ID	Organism	Condition	Number of sequencing reads
DB1	*Bacillus velezensis* E68	Dual culture	6,895,885
DB2	*Bacillus velezensis* E68	Dual culture	5,930,991
DB3	*Bacillus velezensis* E68	Dual culture	2,902,440
DB5	*Bacillus velezensis* E68	Dual culture	4,826,251
DB6	*Bacillus velezensis* E68	Dual culture	3,998,583
Bv1	*Bacillus velezensis* E68	Single culture	7,051,088
Bv2	*Bacillus velezensis* E68	Single culture	6,818,309
Bv3	*Bacillus velezensis* E68	Single culture	5,535,266
Bv5	*Bacillus velezensis* E68	Single culture	10,013,420
Bv6	*Bacillus velezensis* E68	Single culture	10,482,205
DF1	*Fusarium graminearum* DAOMC 180378	Dual culture	23,178,176
DF2	*Fusarium graminearum* DAOMC 180378	Dual culture	24,528,876
DF3	*Fusarium graminearum* DAOMC 180378	Dual culture	20,797,041
DF5	*Fusarium graminearum* DAOMC 180378	Dual culture	21,440,526
DF6	*Fusarium graminearum* DAOMC 180378	Dual culture	23,730,823
Fg1	*Fusarium graminearum* DAOMC 180378	Single culture	18,261,933
Fg2	*Fusarium graminearum* DAOMC 180378	Single culture	18,966,692
Fg3	*Fusarium graminearum* DAOMC 180378	Single culture	19,694,534
Fg5	*Fusarium graminearum* DAOMC 180378	Single culture	19,549,899
Fg6	*Fusarium graminearum* DAOMC 180378	Single culture	16,435,517

**Table 3:** Genes of *B*. *velezensis* E68 and *F*. *graminearum* DAOMC 180378.

	Not significantly differentially expressed genes (% of all expressed genes)	Significantly down-regulated genes (% of all DEGs)	Significantly up-regulated genes (% of all DEGs)	Total expressed genes (% of all expressed genes)
*B*. *velezensis* E68				
Hypothetical	102 (2.86)	31 (2.68)	41 (3.55)	174 (4.88)
Annotated	2308 (64.7)	514 (44.5)	569 (49.2)	3391 (95.1)
Without GO terms	852 (23.9)	153 (13.2)	166 (14.4)	1171 (32.8)
Annotated with GO terms	1558 (43.7)	392 (33.9)	444 (38.4)	2394 (67.2)
Total	2410 (67.6)	545 (47.2)	610 (52.8)	3565 (100)
*F*. *graminearum* DAOMC 180378				
Hypothetical	2490 (25.3)	337 (22.4)	161 (10.7)	2988 (30.4)
Annotated	5838 (59.3)	656 (43.6)	349 (23.2)	6843 (69.6)
Without GO terms	3637 (37.0)	484 (32.2)	246 (16.4)	4367 (44.4)
Annotated with GO terms	4691 (47.7)	509 (33.9)	264 (17.6)	5464 (55.6)
Total	8328 (84.7)	993 (66.1)	510 (33.9)	9831 (100)

Genes were annotated with functional information and gene ontology (GO) terms based on BLASTx searches against the Swiss-Prot database. Genes with minimal expression as determined by edgeR were not included in this table.

### Transcriptomic changes of *B*. *velezensis* in dual culture with *F*. *graminearum*

Following gene ontology enrichment analysis, 16 GO terms were enriched among significantly up-regulated genes in *B*. *velezensis*, whereas 7 GO terms were enriched among significantly down-regulated genes (Table 4). Genes associated to sporulation and related GO terms such as the spore wall and spore germination were up-regulated. Many genes were annotated as spore coat proteins as well as many different genes from sporulation stages II, III, IV and V. These included *spoIIE*, *spoIIGA*, *spoIIR*, and *sigE* from stage II, *spoIID*, *spoIIP*, *spoIIQ*, *spoIIIAH*, *FisB*, *sigG* and *sigK* from stage III, *spoIVB*, *ctpB*, *spoIVA*, *sleB* and *cwlJ* from stages IV and V (S2 Table). The master sporulation regulator gene *spo0A* which initiates sporulation (locus tag GYA98_RS10380) was not significantly differentially regulated (S1 Table). The phosphotransferase *spo0B* (GYA98_RS11640) was also not regulated, but the phosphotransferase *spo0F* (GYA98_RS16270) was down-regulated. The GO term for oxidoreductase activity, acting on CH or CH2 groups, was enriched in up-regulated genes. Genes associated with this term included 2 subunits of ribonucleoside-diphosphate reductase: *nrdE* (GYA98_RS07750) and *nrdF* (GYA98_RS07755) (S1 Table). This enzyme contributes to DNA synthesis by converting ribonucleotides to deoxyribonucleotides. The 4 other up-regulated genes associated to this term were subunits of xanthine dehydrogenase (GYA98_RS17550, 17555, 17560, 17570) (S1 Table). Another subunit GYA98_RS17565 was also up-regulated, though not annotated with the GO term. Xanthine dehydrogenase participates in purine degradation by degrading xanthine into uric acid. Two terms for teichuronic acid biosynthesis were enriched in up-regulated genes (Table 4). All genes associated to these terms were part of the *tua* operon. These genes synthesize teichuronic acid, which replaces teichoic acid in the cell wall in phosphate starvation conditions. In addition, all 5 genes annotated with the ATPase-coupled phosphate ion transmembrane transporter activity GO term were up-regulated. These genes are all part of the PstSABC complex, responsible for phosphate import (GYA98_RS10730, 10735, 10740, 10745, 10750) [46] (S2 Table). Both the *tua* and PstSABC operons are controlled by the Pho regulon. Another member of this regulon, *glpQ* (GYA98_RS00270), was also up-regulated in dual culture [47] (S2 Table).

**Table 4:** Significantly over-represented Gene Ontology (GO) terms following enrichment analysis on up- and down-regulated genes from *B*. *velezensis* E68 and *F*. *graminearum* DAOMC 180378 in dual culture.

Enrichment group	GO term ID	Term	Ontology	False Discovery Rate	DEGs in category	Expressed genes in category
*Bacillus velezensis* E68 up-regulated genes	GO:0032502	developmental process	BP	9.85E-31	122	238
	GO:0048869	cellular developmental process	BP	3.96E-29	114	222
	GO:0030154	cell differentiation	BP	3.96E-29	113	219
	GO:0030435	sporulation resulting in formation of a cellular spore	BP	3.96E-29	113	219
	GO:0048646	anatomical structure formation involved in morphogenesis	BP	3.96E-29	113	219
	GO:0043934	sporulation	BP	1.03E-28	113	221
	GO:0031160	spore wall	CC	5.77E-08	19	24
	GO:0005618	cell wall	CC	9.34E-05	23	44
	GO:0030312	external encapsulating structure	CC	9.34E-05	23	44
	GO:0050845	teichuronic acid biosynthetic process	BP	1.58E-03	7	7
	GO:0050846	teichuronic acid metabolic process	BP	1.58E-03	7	7
	GO:0016725	oxidoreductase activity, acting on CH or CH2 groups	MF	8.02E-03	6	6
	GO:0016020	membrane	CC	9.06E-03	176	758
	GO:0009847	spore germination	BP	1.29E-02	11	18
	GO:0045229	external encapsulating structure organization	BP	3.27E-02	26	71
	GO:0015415	ATPase-coupled phosphate ion transmembrane transporter activity	MF	3.27E-02	5	5
*Bacillus velezensis* E68 down-regulated genes	GO:0016999	antibiotic metabolic process	BP	1.21E-05	44	59
	GO:0017144	drug metabolic process	BP	1.21E-05	44	59
	GO:0017000	antibiotic biosynthetic process	BP	5.11E-05	41	54
	GO:0006099	tricarboxylic acid cycle	BP	4.60E-03	9	11
	GO:0006101	citrate metabolic process	BP	6.31E-03	10	14
	GO:0072350	tricarboxylic acid metabolic process	BP	6.31E-03	10	14
	GO:0000271	polysaccharide biosynthetic process	BP	3.93E-02	15	34
*Fusarium graminearum* DAOMC 180378 up-regulated genes *no GO terms were significantly enriched	GO:0036246	phytochelatin 2 import into vacuole	BP	1.69E-01	3	3
	GO:0071993	phytochelatin transport	BP	1.69E-01	3	3
	GO:0071994	phytochelatin transmembrane transport	BP	1.69E-01	3	3
	GO:0071995	phytochelatin import into vacuole	BP	1.69E-01	3	3
	GO:0071996	glutathione transmembrane import into vacuole	BP	1.69E-01	3	3
*Fusarium graminearum* DAOMC 180378 down-regulated genes	GO:0016491	oxidoreductase activity	MF	2.39E-09	112	614
	GO:0003824	catalytic activity	MF	2.95E-07	326	2802
	GO:0009056	catabolic process	BP	9.19E-05	108	724
	GO:0009712	catechol-containing compound metabolic process	BP	7.04E-04	9	13
	GO:1901575	organic substance catabolic process	BP	7.04E-04	97	665
	GO:0044282	small molecule catabolic process	BP	1.16E-03	38	180
	GO:0018958	phenol-containing compound metabolic process	BP	3.28E-03	10	19
	GO:0042537	benzene-containing compound metabolic process	BP	4.69E-03	11	24
	GO:0016616	oxidoreductase activity, acting on the CH-OH group of donors, NAD or NADP as acceptor	MF	6.32E-03	29	127
	GO:0032787	monocarboxylic acid metabolic process	BP	9.29E-03	39	209
	GO:0016614	oxidoreductase activity, acting on CH-OH group of donors	MF	9.29E-03	34	169
	GO:0046278	3,4-dihydroxybenzoate metabolic process	BP	3.46E-02	5	6
	GO:0016054	organic acid catabolic process	BP	3.46E-02	26	126
	GO:0046395	carboxylic acid catabolic process	BP	3.46E-02	26	126
	GO:0016829	lyase activity	MF	3.53E-02	33	176
	GO:0044248	cellular catabolic process	BP	3.87E-02	77	550

DEGs = differentially expressed genes, BP = biological process, CC = cellular component, MF = molecular function. Genes associated to each GO term are listed in S1 and S2 Tables.

Secondary metabolites in *B*. *velezensis* are produced through enzyme complexes encoded by biosynthetic gene clusters. The transcription of these gene clusters was assessed by treating each cluster as a gene set and using rotation gene set testing (ROAST). The genome of *B*. *velezensis* E68 contains biosynthetic gene clusters which may synthesize 9 known secondary metabolites. Rotation gene set testing showed that all gene clusters were significantly down-regulated, with the exception of surfactin, which was not significant (Table 5). This was also reflected in gene ontology, as GO terms related to antibiotic biosynthesis and metabolism were enriched among down-regulated genes in dual culture (Table 4). Despite being down-regulated, genes responsible for synthesis of secondary metabolites remained fairly highly expressed in dual culture. The median transcripts per million (TPM) of all genes across all dual culture *B*. *velezensis* samples was 73.207. In comparison, the expression of genes annotated as core biosynthetic genes by antiSMASH remained high in down-regulated clusters encoding secondary metabolites (Table 6).

**Table 5:** Rotation gene set testing of biosynthetic gene clusters encoding secondary metabolites in *B*. *velezensis* E68.

Compound	Number of genes	Direction of regulation	False discovery rate
Bacilysin	7	Down	6.79E-08
Macrolactin H	10	Down	2.98E-07
Difficidin	19	Down	3.72E-06
Bacillaene	14	Down	3.72E-06
Bacillibactin	13	Down	6.13E-05
Bacillomycin D	10	Down	4.60E-04
Amylocyclicin	6	Down	1.52E-03
Fengycin	17	Down	0.026
Surfactin	25	Down	0.35

**Table 6:** Normalized expression of core biosynthetic genes from biosynthetic gene clusters encoding secondary metabolites in *B*. *velezensis* E68.

Compound	Core biosynthetic gene locus ID	TPM^$^ in single culture	TPM in dual culture
Surfactin	GYA98_RS00790	41.62444	54.49393
	GYA98_RS00795	39.72677	36.2239
	GYA98_RS00800	10.51762	14.11884
	GYA98_RS00810	49.16629	56.89863
	GYA98_RS00815	55.97234	52.11947
	GYA98_RS00820	77.41527	64.22825
Macrolactin H	GYA98_RS06300	237.6562	90.06079
	GYA98_RS06305	521.1573	100.0418
	GYA98_RS06310	527.2992	96.8625
	GYA98_RS06315	622.4262	113.8966
	GYA98_RS06320	783.2996	181.6299
	GYA98_RS06325	440.9877	118.3611
	GYA98_RS06330	744.2543	223.779
Bacillaene	GYA98_RS07605	410.8624	488.6164
	GYA98_RS07610	420.911	373.5302
	GYA98_RS07615	558.4976	478.7145
	GYA98_RS07625	660.5088	360.3584
	GYA98_RS07640	1183.568	361.8864
	GYA98_RS07645	1198.974	260.8726
	GYA98_RS07650	792.9114	160.4905
	GYA98_RS07655	943.0376	233.3925
	GYA98_RS07660	1074.126	305.5386
Bacillomycin D	GYA98_RS08205	345.918	221.1193
	GYA98_RS08210	604.1638	437.0655
	GYA98_RS08215	565.6975	475.9636
	GYA98_RS08220	277.014	215.3259
Fengycin	GYA98_RS08300	3.060367	33.54516
	GYA98_RS08315	2.122462	36.92852
	GYA98_RS08335	571.6845	177.9275
	GYA98_RS08340	543.9846	173.68
	GYA98_RS08345	693.3596	238.2508
	GYA98_RS08350	463.5909	187.7093
	GYA98_RS08355	580.417	294.5991
	GYA98_RS08365	281.5708	150.397
Difficidin	GYA98_RS10060	1267.013	857.4785
	GYA98_RS10065	1026.037	719.9703
	GYA98_RS10070	899.1106	563.8603
	GYA98_RS10075	1083.195	696.7137
	GYA98_RS10080	901.9802	558.2665
	GYA98_RS10085	912.1075	526.2694
	GYA98_RS10090	1367.896	835.8435
	GYA98_RS10095	1072.906	647.6522
	GYA98_RS10100	1126.655	623.7472
	GYA98_RS10105	510.1765	295.7482
	GYA98_RS10110	953.4498	515.7316
	GYA98_RS10135	573.8718	259.0871
Bacillibactin	GYA98_RS13635	159.2003	49.03839
	GYA98_RS13645	259.1896	116.4149
Amylocyclicin	GYA98_RS13745	25433.92	14390.07
Bacilysin	GYA98_RS16580	1691.767	989.5184

Genes were identified as core biosynthetic genes by antiSMASH.

^$^transcripts per million (TPM)

GO terms related to the tricarboxylic acid (TCA) cycle were also enriched among down-regulated genes. Significantly down-regulated genes associated to these terms included the enzymes of the TCA cycle: 2 citrate synthases (GYA98_RS03820, 12240), aconitase (GYA98_RS08030), isocitrate dehydrogenase (GYA98_RS12235), 2-oxoglutarate dehydrogenase (GYA98_RS08685, 08690), succinate dehydrogenase (GYA98_RS11900, 11905), succinyl-CoA ligase (GYA98_RS07090, 07095) and fumarate dehydrogenase (GYA98_RS14210), as well as a citrate/malate transporter (GYA98_RS17095) (S2 Table). Malate dehydrogenase (GYA98_RS12230), which was not annotated with the TCA cycle GO terms, was also significantly down-regulated. The GO term for polysaccharide biosynthetic process was also enriched among the down-regulated genes. Of the significantly down-regulated genes associated to this term, 14 of the 15 genes of the *eps* operon (*epsB-O*, GYA98_RS14870-14935) were included (S2 Table). The *epsA* homolog in *B*. *velezensis* E68 (GYA98_RS14940) was not significantly differentially expressed. The *eps* operon encodes enzymes responsible for synthesis of exopolysaccharide, a component of the extracellular biofilm matrix [48, 49]. In addition, *tasA* (GYA98_RS10575), the major protein component of the extracellular matrix, was also down-regulated in dual culture, though it wasn’t annotated with GO terms.

### Transcriptomic changes of *F*. *graminearum* in dual culture with *B*. *velezensis*

In *F*. *graminearum*, no GO terms were significantly enriched among up-regulated genes in dual culture. Despite not being significantly enriched, 5 GO terms had all 3 of their associated genes be up-regulated in *F*. *graminearum* (Table 4). FGRAMPH1_01G11895 and FGRAMPH1_01G06603, both annotated as heavy metal tolerance proteins which may transport phytochelatin, and FGRAMPH1_01G26673, an ATP-binding cassette transporter, were all up-regulated in dual culture conditions and were the only genes associated with the 5 GO terms for phytochelatin transport/import and glutathione import into the vacuole (S3 Table). The relative lack of total genes associated to the terms prevented these terms from being significantly enriched after correction for multiple testing. The genome of *F*. *graminearum* DAOMC 180378 was annotated with 4 genes described as killer protein 4-like. All 4 genes were up-regulated in dual culture (S4 Table).

In the 16 GO terms enriched among down-regulated genes in *F*. *graminearum*, many were related to the broad categories of the catabolic process term and the oxidoreductase activity term. More specifically, many of the terms related to the metabolism of aromatic compounds, notably the terms for phenol containing, benzene-containing and catechol-containing compound metabolic processes, as well as the term for 3,4-dihydroxybenzoate (also known as protocatechuate) metabolic process (Table 4). Significantly down-regulated genes associated to these compounds included genes upstream of protocatechuate/catechol: 2 genes annotated as quinate dehydrogenases (FGRAMPH1_01G13935 and FGRAMPH1_01G18555), one gene annotated either as a quinate or shikimate dehydrogenase (FGRAMPH1_01G13945), 2,3-dihydroxybenzoate decarboxylase (FGRAMPH1_01G28015) and a 3-dehydroshikimate dehydratase (FGRAMPH1_01G08441) (S3 Table). In addition, genes degrading catechol and protocatechuate were down regulated: a catechol 1,2-dioxygenase (FGRAMPH1_01G13423) and another gene annotated as a catechol 1,2-dioxygenase, but a BLAST search against the DAOMC 180378 genome showed that protocatechuate-3,4-dioxygenase from *Aspergillus nidulans* was homologous to this gene (FGRAMPH1_01G12667) [50]. Also part of the significantly down-regulated genes associated to benzene compounds were 3 genes from the homogentisate pathway, which utilizes phenylacetate and phenylalanine as carbon sources. Homogentisate 1,2-dioxygenase (FGRAMPH1_01G05639), 3-hydroxyphenylacetate 6-hydroxylase (FGRAMPH1_01G22299) and fumarylacetoacetase (FGRAMPH1_01G11459) were all down-regulated (S3 Table). In addition, 3 genes from the kynurenine pathway were down-regulated: 2 kynureninases (FGRAMPH1_01G16409 and FGRAMPH1_01G14561), as well as an indoleamine 2,3-dioxygenase (FGRAMPH1_01G16407) (S3 Table). This pathway degrades tryptophan into NAD+.

In dual culture, *F*. *graminearum* hyphae was distorted and swelling occurred, based on microscopy (Fig 3). However, the differential gene expression data from RNA-seq did not show concerted differential regulation of genes relating to this phenotype. This included genes related to ergosterol synthesis, glucan or chitin synthases and genes related to autophagy. Similarly, *F*. *graminearum* also did not show large scale differential regulation of genes relating to oxidative stress, specifically genes encoding for superoxide dismutases and catalases (S4 Table). Genes annotated as glutathione S-transferases were also mostly not differentially expressed, though some were significantly down-regulated in dual culture (S4 Table).

*F*. *graminearum* DAOMC 180378 is known to produce deoxynivalenol, a trichothecene mycotoxin [37]. To assess the effect of dual culture with *B*. *velezensis* on trichothecene mycotoxin expression, genes from the core trichothecene gene cluster, as well as other genes from the trichothecene pathway were evaluated for differential expression (Table 7) [51]. Biosynthetic enzymes *TRI3*, *TRI8* and *TRI11* did not show significant differential expression. The biosynthetic enzymes *TRI1*, *TRI5*, *TRI13* and *TRI101* were significantly down-regulated, while *TRI4* was down-regulated, only approaching significance. The accessory proteins *TRI6*, *TRI9*, *TRI10*, *TRI11* and *TRI14* did not show significant differential expression. The transcription factor *TRI15* showed significant up-regulation in dual culture. *TRI15* is likely a negative regulator of trichothecene expression [52].

**Table 7:** Fold change of expression of trichothecene pathway genes in *F*. *graminearum* DAOMC 180378 in dual culture with *B*. *velezensis* E68.

Locus ID	Gene	Log_2_ fold change	False discovery rate	Function
FGRAMPH1_01G13947	*TRI15*	3.03529	3.72E-06	Transcription factor, negative regulator
FGRAMPH1_01G00223	*TRI1*	-1.06495	0.014269	Biosynthetic enzyme
FGRAMPH1_01G13121	*TRI13*	-2.08402	0.021416	Biosynthetic enzyme
FGRAMPH1_01G25923	*TRI101*	0.982818	0.027902	Biosynthetic enzyme
FGRAMPH1_01G13111	*TRI5*	-1.19642	0.028048	Biosynthetic enzyme
FGRAMPH1_01G13107	*TRI4*	-1.03885	0.053723	Biosynthetic enzyme
FGRAMPH1_01G13101	*TRI8*	-0.9157	0.176208	Biosynthetic enzyme
FGRAMPH1_01G13123	*TRI14*	-0.84547	0.185704	Virulence factor, unknown function
FGRAMPH1_01G13105	*TRI3*	0.889523	0.345196	Biosynthetic enzyme
FGRAMPH1_01G13115	*TRI9*	-0.54126	0.751079	Unknown function
FGRAMPH1_01G13109	*TRI6*	-0.52321	0.778677	Transcription factor
FGRAMPH1_01G13113	*TRI10*	0.359458	1	Transcription factor
FGRAMPH1_01G13117	*TRI11*	-0.36532	1	Biosynthetic enzyme
FGRAMPH1_01G05631	*TRI12*	-0.15582	1	Transporter

Functions were assigned to genes based on [51].

### Validation of RNA-seq via quantitative PCR

**Fig 4:**
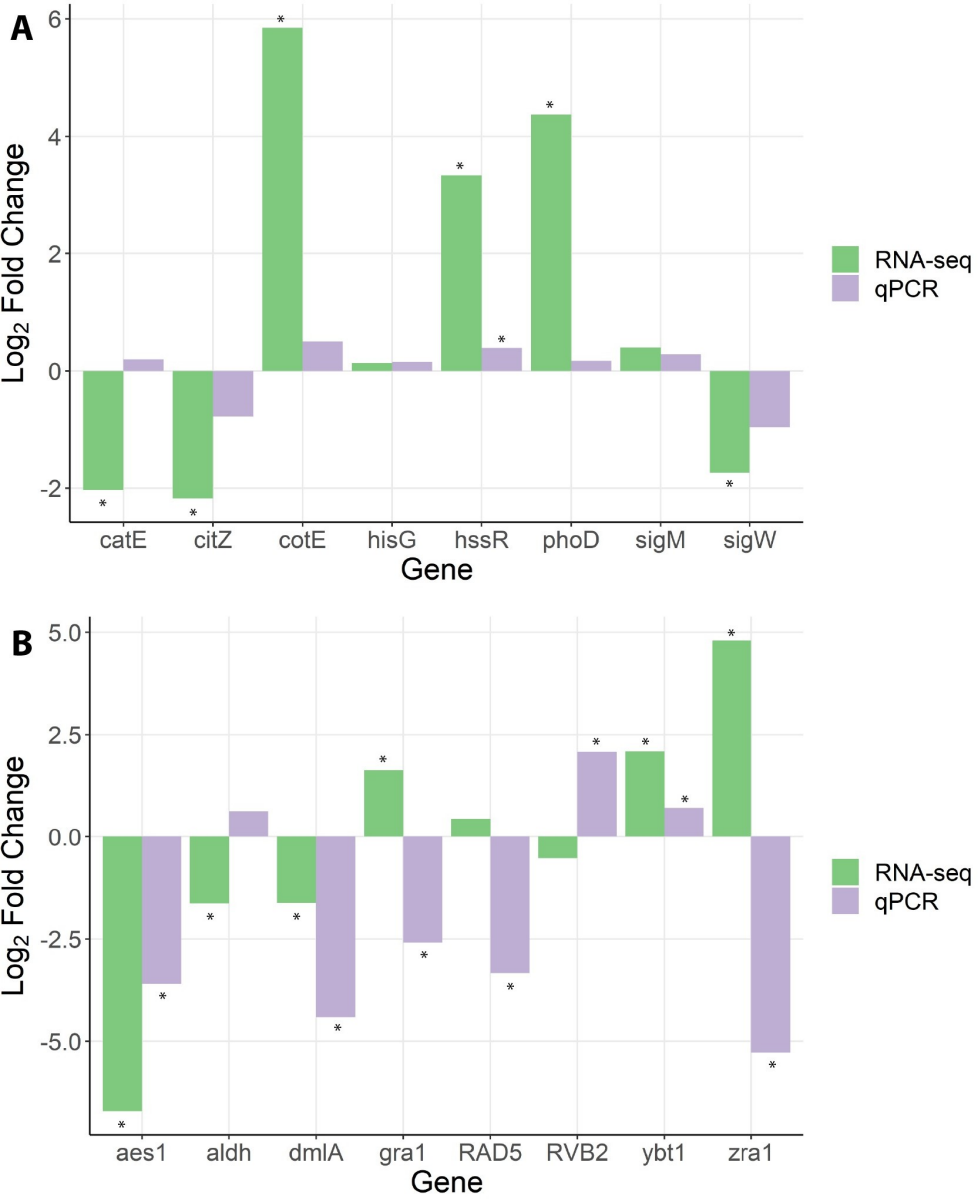
Log_2_ fold change of selected genes between single and dual culture based on RNA-seq and qPCR for (A) *B*. *velezensis* E68 and (B) *F*. *graminearum* DAOMC 180378. Asterisks (*) indicate significantly differentially expressed genes for RNA-seq and indicate p < 0.05 for qPCR.

In order to validate the results of RNA-seq, 8 genes were selected per organism to evaluate their differential expression through qRT-PCR (Table 1). Three RNA samples per condition were used for analysis (Bv1, Bv2, Bv5, DB1, DB2, DB5, Fg1, Fg2, Fg5, DF1, DF2, DF5). The housekeeping genes were selected based on use in previous studies as well as lack of differential expression in the RNA-seq dataset. In *B*. *velezensis* E68, the 2 selected non-differentially expressed genes (*hisG* and *sigM*) saw identical results in qRT-PCR (Fig 4A). The gene *hssR* showed significant up-regulation in both RNA-seq and qRT-PCR. Four significantly differentially expressed genes (*phoD*, *sigW*, *cotE*, *citZ*) had similar trends between RNA-seq and qRT-PCR, though the qRT-PCR fold-changes were not significant after t-test. One gene, *catE* showed opposite trends in qPCR, though the result was not significant. In *F*. *graminearum* DAOMC 180378, the 2 selected non-differentially expressed genes *RVB2* and *RAD5* showed significant differential expression using qRT-PCR (Fig 4B). The 3 genes *YBT1*, *AES1* and *DMLA* showed significant differential expression in the same direction in both RNA-seq and qRT-PCR. The genes *ZRA1* and *GRA1* showed significant regulation in opposite directions in RNA-seq and qRT-PCR. The *ALDH* gene also showed opposite regulation by RNA-seq and qRT-PCR, though the qRT-PCR result was not significant.

## Discussion

A dual RNA-seq approach was developed to compare the transcriptional profiles of two organisms, *Bacillus velezensis* E68 and *Fusarium graminearum* DAOMC 180378 in single and dual culture. Previously, dual RNA-seq was employed to investigate the interaction between the rhizobacteria *Lysobacter capsici* and the oomycete *Phytophthora infestans* [53]. In addition, a dual microarray approach was used to assess the transcriptional changes from both sides of a *Bacillus subtilis* and *Aspergillus niger* interaction [54]. However, to the best of our knowledge, this is the first time a dual RNA-seq approach has been used to study the transcriptional changes between a biocontrol candidate bacteria and a filamentous fungus. Strains of *B*. *velezensis* and the closely related *B*. *subtilis* were previously shown to inhibit growth of *F*. *graminearum* on solid media, demonstrating the potential of these strains as biological control candidates [23, 24, 55]. In our study, *B*. *velezensis* E68 showed very similar inhibition of *F*. *graminearum* DAOMC 180378. Microscopic observations of *F*. *graminearum* exposed to *B*. *velezensis* showed distorted hyphal tips and circular swellings, most likely due to secretion of antifungal lipopeptides by *B*. *velezensis*. This phenotype was also observed in other studies exposing *F*. *graminearum* to both *B*. *velezensis* cultures and also purified lipopeptide extracts from *B*. *velezensis* and *B*. *subtilis* [23, 56–59]. Lipopeptides secreted from *B*. *velezensis* and similar species are known to cause damage to the plasma membrane and cell wall of various fungal phytopathogens, including *F*. *graminearum* [23, 58, 60, 61].

When confronted with *F*. *graminearum*, *B*. *velezensis* showed clear up-regulation of many genes related to sporulation. Strains of *Bacillus* tend to sporulate under conditions of limited nutrient availability, though the specific signals by which this is determined are unknown [62]. In our study, genes related to stages 0 and I of sporulation did not show significant differential expression in *B*. *velezensis*. This includes the master regulator of sporulation *spo0A*, though since its activity is reliant on its phosphorylation state, its transcriptional status may not be of importance [63]. However, multiple genes from stages II through V were up-regulated. SigF, the first sigma factor related to sporulation is produced prior to stage II. The gene product of *spoIIE* is responsible for activating this sigma factor in the forespore [64]. Consistent with this idea, in our study *spoIIE* was up-regulated in *B*. *velezensis* E68 in dual culture. In addition, *spoIIGA* and *spoIIR*, the activators of *sigE*, as well as *sigE* itself, were all up-regulated in dual culture, triggering stage III: engulfment [65]. Genes required for engulfment and membrane fission were up-regulated in dual culture: *spoIID*, *spoIIP*, *spoIIQ*, *spoIIIAH* and *FisB* [66, 67]. The forespore-specific sigma factor *sigG* and the mother cell specific sigma factor *sigK* are both activated at the end of engulfment, these are both up-regulated in dual culture. In addition the *sigK* activators *spoIVB* and *ctpB* were up-regulated [68–70]. Many spore cortex coat proteins related to stages IV and V were up-regulated, including *spoIVA*, which forms the base layer of the spore coat [68]. Furthermore, spore germination genes were also up-regulated in dual culture. *sleB* and *cwlJ* are enzymes which lyse peptidoglycan during germination and were both up-regulated. However, they lie dormant in the spore in a mature form [71]. Taken together, these results suggest that in the presence of *F*. *graminearum*, *B*. *velezensis* cells sporulate, at stages II through V of sporulation on day 3 during our dual culture assay. Previous research showed *B*. *subtilis* up-regulated late sporulation genes when grown on *Aspergillus niger* hyphae, though no significant difference in spore content was found [54].

In dual culture, *B*. *velezensis* E68 down-regulated key components of the extracellular biofilm matrix: the *eps* operon and the *tasA* gene [72]. The decision to begin biofilm formation and the decision to sporulate are both governed by the Spo0A protein [73]. Spo0A is a transcriptional regulator whose activity depends on its phosphorylation state. High levels of phosphorylated Spo0A promote entry into sporulation through repression of *sinI* expression [63]. Research also shows that sporulation may require biofilm formation to occur [73]. As a result, the down-regulation of biofilm components in *B*. *velezensis* coincides with the up-regulation of sporulation genes.

The genes encoding for the tricarboxylic acid cycle enzymes were down-regulated in *B*. *velezensis* in dual culture. This is consistent with previous research studying the transcriptomics of the life cycle of *Bacillus pumilus* [74]. *B*. *pumilus* showed decreased expression of TCA cycle genes in the stationary growth phase as compared to the exponential growth phase, in addition to an increase in expression of sporulation-related genes, much the same as *B*. *velezensis* in our experiment. Furthermore, TCA cycle genes were down-regulated and sporulation genes were up-regulated in *B*. *subtilis* which were attached to *Aspergillus niger* hyphae as compared to planktonic cells [54].

Secondary metabolism in *B*. *velezensis* E68 was evidently altered, with all known biosynthetic gene clusters (with the exception of surfactin) showing reduced expression in dual culture with *F*. *graminearum*. Despite the down-regulation, these metabolites remained expressed in dual culture and likely secreted into the media, as reflected by the changes in fungal hyphae appearance in dual culture (Fig 3). This down-regulation is unexpected, as multiple studies of lipopeptide-secreting *Bacillus* report up-regulation of lipopeptide related genes or increased lipopeptide concentrations in confrontation with fungi [75–79]. However, surfactin was found to be down-regulated in *B*. *subtilis* cells attached to hyphae of *A*. *niger* [54]. Our experiment was performed at a single time-point: 3 days after establishment of interaction. It may be possible that different regulation patterns could be expressed for secondary metabolism genes in earlier or later stages of the interaction. Using proteomic methods for the detection of lipopeptides and other secondary metabolites could also show variations from the gene expression data obtained here.

In dual culture, *B*. *velezensis* also showed signs of phosphate stress. In times of phosphate starvation, *Bacillus* strains induce genes of the Pho regulon, controlled by the PhoP sensor and the PhoR regulator, though these were not differentially expressed in our study [47]. When confronted with *F*. *graminearum*, *B*. *velezensis* up-regulated members of the Pho regulon, such as the *tua* operon. This operon synthesizes teichuronic acid, a component of the cell wall. In phosphate starvation conditions, teichuronic acid replaces teichoic acid in the cell wall [80]. Phosphate can then be collected from the teichoic acid through the action of *phoD*, which was also up-regulated in dual culture [81]. The PstSABC operon is another part of the Pho regulon which was up-regulated in dual culture. The PstSABC complex is a phosphate importer which may also play a role in sensing extracellular phosphate concentrations through interaction with PhoR [46, 82]. Furthermore, *B*. *velezensis* up-regulated *glpQ*, also part of the Pho regulon. This gene encodes a glycerophosphoryl diester phosphodiesterase, which hydrolyzes deacetylated phospholipids [83].

Taken together, these results indicate that in dual culture with *F*. *graminearum*, *B*. *velezensis* E68 entered sporulation, as shown by the up-regulation of sporulation and biofilm formation genes. This could be due to different stresses, including phosphate starvation. Consistent with its entry into sporulation, *B*. *velezensis* down-regulated genes related to carbohydrate metabolism and secondary metabolite biosynthesis.

In our study, there were no GO terms enriched among up-regulated genes in *F*. *graminearum*. This can be partially attributed to the low number of expressed genes annotated with GO terms (55.6% of expressed genes). Additionally, only 510 genes were significantly up-regulated, as compared to 993 down-regulated genes. As a result, global up-regulation of particular molecular functions or biological processes was not found. When the fungal phytopathogen *Sclerotinia sclerotiorum* was confronted to *B*. *velezensis* fermentation broth, the fungus was found to induce genes related to cell wall and ergosterol synthesis, fatty acid synthesis, antioxidants and autophagy [84]. In this study, genes related to cell wall synthesis via either glucan synthase or chitin synthase were mostly not differentially expressed, with chitin synthase 6 (FGRAMPH1_01G22563) being the only significantly up-regulated chitin synthase and chitin synthase 5 (FGRAMPH1_01G10489) being down-regulated. Distinct up-regulation of ergosterol pathway genes as defined through annotation or previous evidence was also not found. It may be that fungal cells most affected by the bacterial lipopeptides were lysed and their RNA could not be sequenced. Genes related to autophagy were also not differentially expressed in dual culture [85]. In addition to damage to the cell wall and membranes, *Bacillus* lipopeptides can also induce accumulation of reactive oxygen species [58, 86]. However, our data signalled that *F*. *graminearum* did not show concerted up-regulation of genes relating to oxidative stress. Five genes previously identified as superoxide dismutases did not show differential expression [87]. From a list of 7 previously identified catalases in *F*. *graminearum*, 6 showed no differential expression, while 1 was significantly down-regulated [88]. One catalase which was separately identified using its annotation was up-regulated in dual culture (FGRAMPH1_01G05343). In addition, there were no genes annotated as glutathione S-transferase that were up-regulated in dual culture, though several were significantly down-regulated. *F*. *graminearum* did show up-regulation of 3 genes related to heavy metal tolerance through phytochelatin or glutathione transport into the vacuole. Phytochelatin is a polymer of glutathione which is used to sequester cadmium. Upon transport into the vacuole via HMT1, an ABC transporter, the phytochelatin-cadmium complex can integrate sulfide to achieve higher cadmium binding and stability. Phytochelatin may also confer tolerance to copper, mercury, silver and arsenate ions [89].

While not associated to particular GO terms, all 4 genes previously identified as killer protein 4-like proteins were up-regulated in dual culture. These had previously been shown to be induced by *F*. *graminearum* in stressful conditions such as high salt concentrations or osmotic pressures, and it is believed they play a role in virulence, though it is uncertain which specific function they perform in the stress response [90].

*F*. *graminearum* showed a clear down-regulation of genes associated to metabolism of phenolic compounds in dual culture with *B*. *velezensis*. In nature, *F*. *graminearum* overwinters on infested crop residues, adopting a saprotrophic lifestyle [91]. Fungi living on plant litter must decompose the lignin-related aromatic compounds to use as a carbon source [92]. Fungi employ various pathways to degrade and recycle aromatic compounds, usually through one of 5 intermediates: protocatechuate, catechol, homogentisate, gentisate or hydroquinone [50]. Potato, a major component of the culture media, is known to contain various phenolic compounds such as phenolic acids and lignin [93, 94]. *F*. *graminearum* showed down-regulation of genes in the protocatechuate, catechol and homogentisate pathways. It also down-regulated genes of the kynurenine pathway, responsible for degrading the aromatic amino acid tryptophan into NAD+. It is possible that in dual culture, *B*. *velezensis* is metabolizing some of the aromatics in the media, lessening the requirements for aromatic metabolism in the fungus. Phenolic compounds such as 2,3-dihydroxybenzoate are required for the synthesis of bacillibactin, a secondary metabolite produced by *B*. *velezensis* [95]. This notion merits further investigation.

In dual culture, *F*. *graminearum* showed significant down-regulation of 3 biosynthetic enzymes: TRI1, TRI5 and TRI13, with TRI4 approaching significance. TRI1, TRI4 and TRI5 are all key components of the trichothecene synthesis pathway [51]. TRI4 and TRI5 carry out the first 5 steps of the biosynthetic pathway, while TRI1 performs a later step. TRI101 was significantly up-regulated in dual culture, however, which is unexpected given its position between TRI4 and TRI1 in the biosynthetic pathway. TRI13 has no active functionality in deoxynivalenol-producing chemotypes of *F*. *graminearum*, which include strain DAOMC 180378 [51]. This is reflected by its low expression relative to the other genes of the TRI locus. TRI15 is a negative regulator of trichothecene biosynthesis which was up-regulated in dual culture [52]. Its specific gene targets are unknown, though exogenously added trichothecenes induced its expression in *F*. *sporotrichoides*, a trichothecene producer. Studies of trichothecene gene expression have not analyzed TRI15 expression, though we speculate its up-regulation in dual culture caused down-regulation of trichothecene biosynthetic genes. Down-regulation of trichothecene biosynthetic genes or reduction in trichothecene production through dual culture with *Bacillus* has been observed before [22–24]. The mechanism through which this gene inhibition occurs remains to be elucidated.

In summary, this work investigated the transcriptomic response of both a biocontrol candidate bacteria and its fungal phytopathogenic target in a dual culture assay. *B*. *velezensis* E68 was found to up-regulate genes related to sporulation and phosphate stress response and down-regulate genes related to secondary metabolism, biofilm formation and the TCA cycle. *F*. *graminearum* DAOMC 180378 saw up-regulation of heavy metal tolerance genes and killer protein-4 like proteins. It down-regulated trichothecene biosynthesis and phenol metabolism.

## Supporting information

S1 Table Differential gene expression data for expressed genes of *Bacillus velezensis* E68. (XLSX)

S2 Table Subsets of S1 Table according to biological function. (XLSX)

S3 Table Differential gene expression data for expressed genes of *Fusarium graminearum* DAOMC 180378. (XLSX)

S4 Table Subsets of S3 Table according to biological function. (XLSX)

S1 Fig *Bacillus velezensis* strain E68 inhibits growth of various fungal phytopathogens. Fungal plugs from 7–10 day old cultures on PDA were placed at the centre of an LBA (A-H) or PDA (I-P) plate. On interaction plates (left) *B*. *velezensis* strain E68 was grown overnight in LB media at 37°C and diluted to OD_600_ of 1.0. 10 μL of cell suspension was dropped 2 cm from the fungal agar plug. Fungal cultures were grown alone on control plates (right). Plates were incubated at 25°C for 4 days and then imaged. (A-B) *Rhizoctonia solani* AG-4, (C-D) *Rhizoctonia solani* AG-1-1A, (E-F) *Fusarium solani*, (G-H) *Fusarium oxysporum*, (I-J) *Fusarium graminearum*, (K-L) *Rhizoctonia solani* AG-3, (M-N) *Fusarium proliferatum*, (O-P) *Chaetomium globosum*.
(TIF)
