# Supplementary figures and images for: Comparative transcriptome analysis reveals the biocontrol mechanism of Bacillus velezensis E68 against Fusarium graminearum DAOMC 180378, the causal agent of Fusarium head blight
Source: PLoS One. 2023 Jan 26;18(1):e0277983. doi: 10.1371/journal.pone.0277983 (PMC9879434; doi:10.1371/journal.pone.0277983)

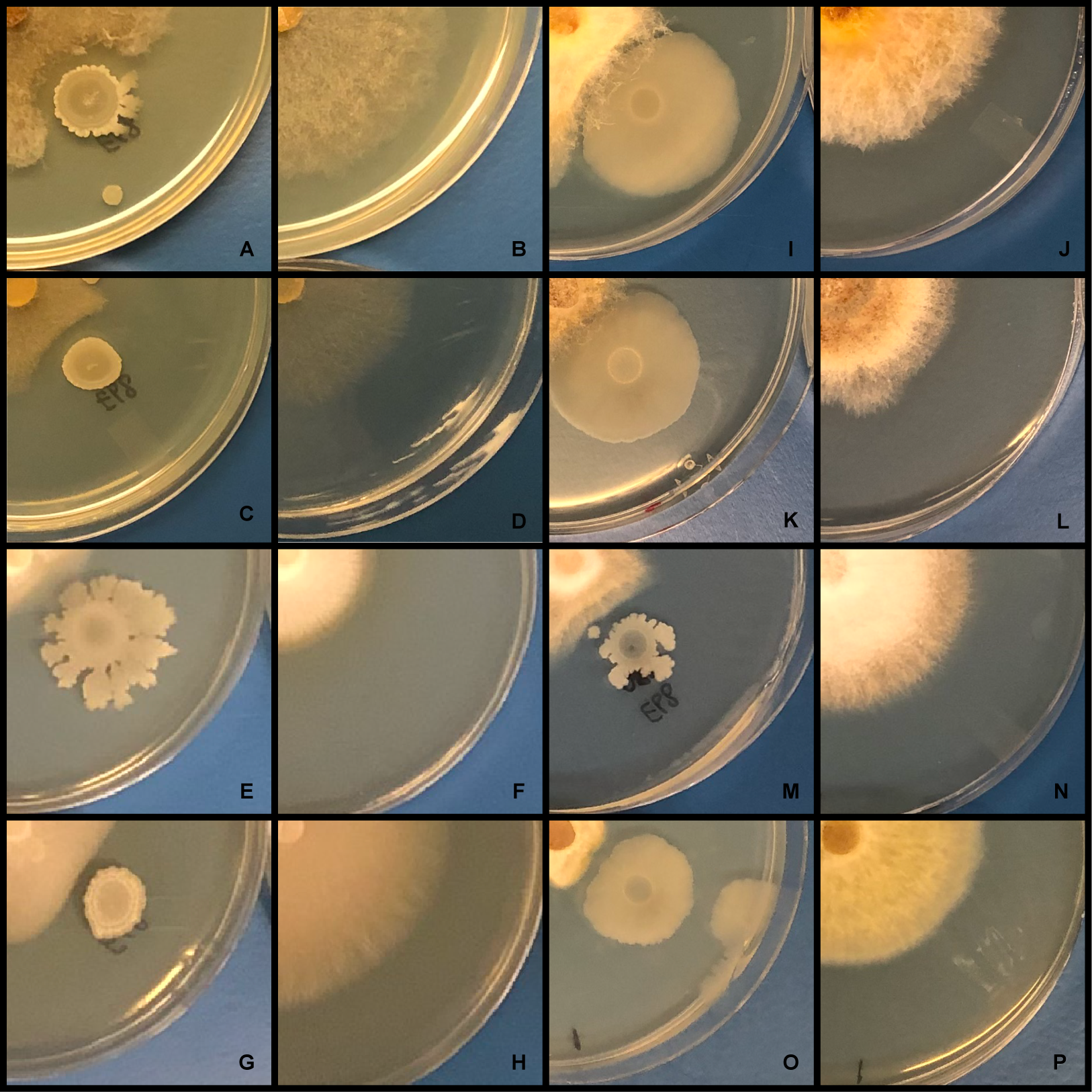

Supplement: S1 Fig — Fungal plugs from 7–10 day old cultures on PDA were placed at the centre of an LBA (A-H) or PDA (I-P) plate. On interaction plates (left) B. velezensis strain E68 was grown overnight in LB media at 37°C and diluted to OD600 of 1.0. 10 μL of cell suspension was dropped 2 cm from the fungal agar plug. Fungal cultures were grown alone on control plates (right). Plates were incubated at 25°C for 4 days and then imaged. (A-B) Rhizoctonia solani AG-4, (C-D) Rhizoctonia solani AG-1-1A, (E-F) Fusarium solani, (G-H) Fusarium oxysporum, (I-J) Fusarium graminearum, (K-L) Rhizoctonia solani AG-3, (M-N) Fusarium proliferatum, (O-P) Chaetomium globosum. (TIF) [file pone.0277983.s005.tif]
